# Noncoding Subgenomic Flavivirus RNA: Multiple Functions in West Nile Virus Pathogenesis and Modulation of Host Responses

**DOI:** 10.3390/v6020404

**Published:** 2014-01-28

**Authors:** Justin A. Roby, Gorben P. Pijlman, Jeffrey Wilusz, Alexander A. Khromykh

**Affiliations:** 1 Australian Infectious Disease Research Centre, School of Chemistry and Molecular Biosciences, The University of Queensland, St. Lucia, Brisbane 4072, Australia; E-Mail: j.roby@uq.edu.au; 2 Laboratory of Virology, Wageningen University, Wageningen, 6708NW, The Netherlands; E-Mail: gorben.pijlman@wur.nl; 3 Department of Microbiology, Immunology & Pathology, Colorado State University; Fort Collins, CO 80523, USA; E-Mail: jeffrey.wilusz@colostate.edu

**Keywords:** flavivirus, sfRNA, replication, pathogenicity, interferon, RNAi, XRN1

## Abstract

Flaviviruses are a large group of positive strand RNA viruses transmitted by arthropods that include many human pathogens such as West Nile virus (WNV), Japanese encephalitis virus (JEV), yellow fever virus, dengue virus, and tick-borne encephalitis virus. All members in this genus tested so far are shown to produce a unique subgenomic flavivirus RNA (sfRNA) derived from the 3' untranslated region (UTR). sfRNA is a product of incomplete degradation of genomic RNA by the cell 5'–3' exoribonuclease XRN1 which stalls at highly ordered secondary RNA structures at the beginning of the 3'UTR. Generation of sfRNA results in inhibition of XRN1 activity leading to an increase in stability of many cellular mRNAs. Mutant WNV deficient in sfRNA generation was highly attenuated displaying a marked decrease in cytopathicity in cells and pathogenicity in mice. sfRNA has also been shown to inhibit the antiviral activity of IFN-α/β by yet unknown mechanism and of the RNAi pathway by likely serving as a decoy substrate for Dicer. Thus, sfRNA is involved in modulating multiple cellular pathways to facilitate viral pathogenicity; however the overlying mechanism linking all these multiple functions of sfRNA remains to be elucidated.

## 1. Conservation of sfRNA in the Genus *Flavivirus*

### 1.1. Genus *Flavivirus*

**Figure 1:**
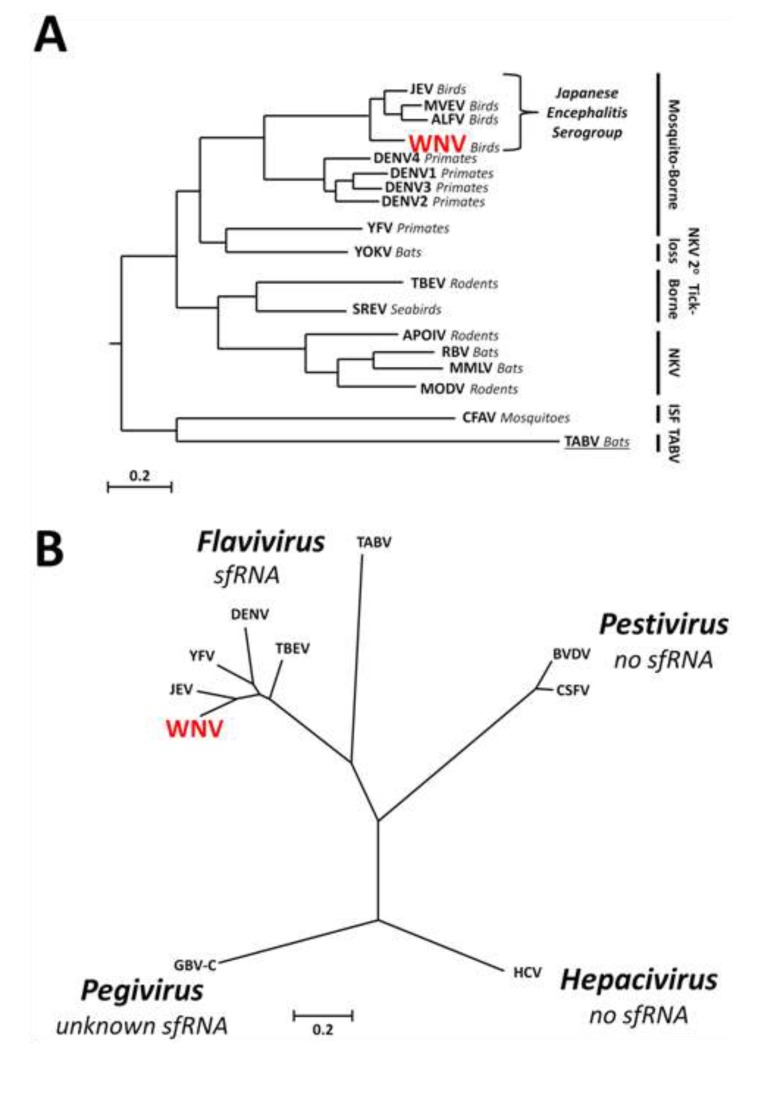
Phylogenetic relationships and sfRNA production within the genus *Flavivirus* and the family Flaviviridae. (**A**) Phylogenetic tree demonstrating the evolutionary relationships between flaviviruses characterised for sfRNA production and basal Tamana bat virus (TABV) which remains uncharacterised (underlined). The tree was generated using the amino acid sequence of the entire genomic polyproteins. Vector classes for virus groups are indicated by black bars to the right of the tree. Primary vertebrate hosts are indicated in italics (other than for ISFs). Bar = 0.2 substitutions per site. ISF = insect-specific flavivirus; NKV = no known vector. Modified from Cook *et al.*, 2012 and Kitchen *et al.*, 2011 [5,6]; (**B**) sfRNA does not appear to be produced by other genera (*Hepacivirus*, *Pestivirus*) within the family Flaviviridae. The proposed genus *Pegivirus* remains uncharacterised for sfRNA production. Phylogenetic tree generated using the amino acid sequences of viral RNA-dependant RNA polymerases. Bar = 0.2 substitutions per site. Modified from Stapleton *et al.*, 2011 [11].

West Nile virus (WNV) is an emerging pathogen of humans that has the capacity to induce fatal encephalitis in the infected host [1,2,3]. WNV is a member of the genus *Flavivirus* within the family Flaviviridae which comprises small, enveloped viruses with non-segmented genomes consisting of single-stranded positive sense RNA. The *Flavivirus* genus can be divided into four main groups: mosquito-borne, tick-borne, no known vector (NKV), and the highly divergent insect-specific (ISF) groups (Figure 1A,B) [4,5,6,7,8]. Viruses belonging to the mosquito- and tick-borne groups are maintained in a natural transmission cycle between amplifying vertebrate hosts and particular haematophagous arthropods. NKV viruses are restricted to mammalian bat or rodent hosts, without a known arthropod vector for transmission. ISFs are the most divergent group, infecting and transmitted by predominantly *Aedes* and *Culex* genus mosquitos, and are unable to infect vertebrate hosts. Tamana bat virus (TABV) is the most divergent species apparently replicating exclusively in mammalian cells [9], requiring placement in its own group removed from the rest of the genus [10,11].

### 1.2. The 3′UTR of the Family Flaviviridae

The family Flaviviridae is composed of viruses with monocistronic single-stranded positive-polarity RNA genomes that lack a polyA tail. Flanking the single ORF are highly structured 5' and 3' untranslated regions (UTRs) that act as important mediators of viral genome replication and translation. These UTRs contain conserved and highly elaborated secondary structures that are generally more pronounced at either end of the genome [12]. Viruses of the genera *Hepacivirus*, *Pegivirus*, and *Pestivirus* concentrate the highest degree of structural elaboration at their 5'UTRs which have evolved to function as internal ribosome entry sites (IRES) that drive cap-independent translation of the polyprotein. Members of the genus *Flavivirus*, to which WNV belongs, retain an m^7^GpppAmpN_1_ cap structure at the 5'-terminus and thus have shorter 5'UTRs (≈100 nt) [13] consisting of a pair of conserved stem-loops (SL-A and SL-B) [14] and encoding regions important for genome cyclisation at the initial phase of replication (5'CS, 5'UAR, and 5'DAR; reviewed in [15]).

**Figure 2:**
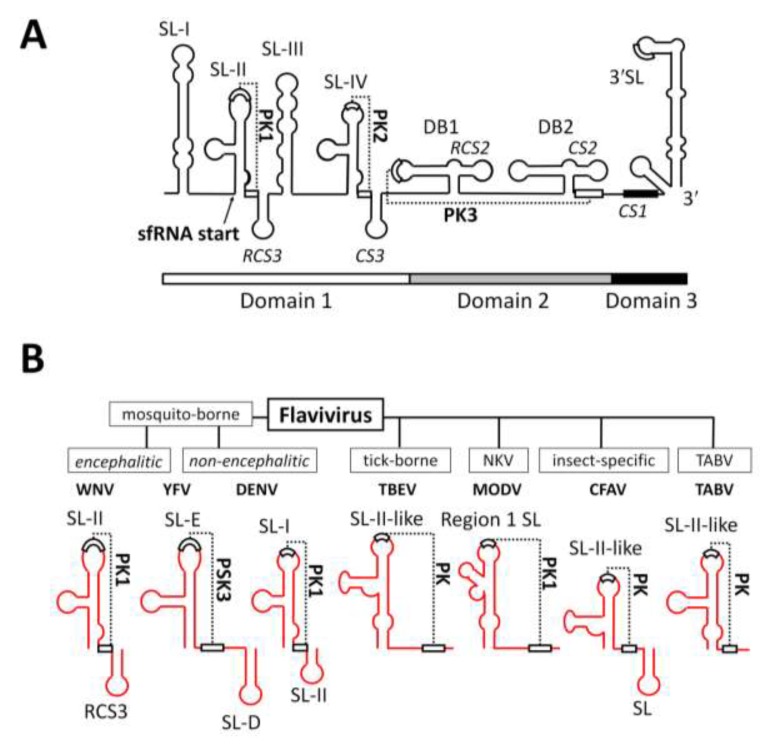
Structural elements within the 3'UTR of WNV and conservation of SL-II within the genus *Flavivirus*. (**A**) Schematic representation of the 3'UTR of WNV demonstrating the arrangement of stem-loops (SL) and pseudoknots (PK) and the sfRNA 5'-terminus. Modified from Pijlman *et al.*, 2008 [18] and Funk *et al.*, 2010 [19]; (**B**) Schematic representation demonstrating the predicted conservation of the SL-II/PK1-like RNA structure within the 3'UTR of divergent members of the genus *Flavivirus*.

The 3'UTR of flaviviruses is considerably larger (≈380–600 nt in length) and can—in the case of mosquito-borne flaviviruses—be divided into three domains: a highly variable proximal domain 1 that directly follows the stop codon, a second domain 2 with moderately conserved sequence and a number of stem-loop and dumbbell structures, and the highly conserved distal domain 3 which contains the complementary cyclisation elements and the stable, terminal stem-loop structure (Figure 2A) [15]. The 3′UTR of WNV consists of an AU-rich stem-loop SL-I structure followed by a highly-conserved, branched, pseudoknot (PK1)-forming SL-II immediately preceding a short conserved loop RCS3 (Figure 2A). This structural motif is then effectively repeated by SL-III, SL-IV (with PK2) and CS3 (Figure 2A). Downstream of this are two dumbbell structures DB1 and DB2, with DB1 also predicted to form pseudoknot PK3 (Figure 2A). This region is then followed by a predicted short stem-loop and the large terminal 3'SL (Figure2A) [15,16,17,18].

The 3'UTR of flaviviruses has been demonstrated to bind to several host proteins (see the last section) as well as proteins of the viral replication complex (RC). Such interactions promote genomic RNA cyclisation which is important for two distinct functions: (i) SL-A in the 5'UTR acts as a promoter element [20,21,22] that stimulates the viral RNA-dependent RNA polymerase NS5 to begin negative strand synthesis at the 3'UTR, initiating the replication cycle [15], and (ii) the 3'UTR binds to host proteins that normally associate with mRNA polyA tails such as phosphorylated translation elongation factor 1α (EF-1α) and poly(A)-binding protein (PABP), thus bringing the 5' and 3'UTRs into close association, which allows cap-dependent translation of the viral polyprotein to proceed [15,23,24,25].

### 1.3. Discovery of sfRNA

The accumulation of a small viral RNA species now known as sfRNA was first observed for the mosquito-borne encephalitic flaviviruses Murray Valley encephalitis virus (MVEV) [26], Japanese encephalitis virus (JEV) [27], and WNV [28]. These early observations identified the presence of the sfRNA species *in vitro* and *in vivo* without ascribing a biological role to the moiety, although in the JEV paper [27] the authors mapped sfRNA to the 3′UTR and determined its 5' end. Although attempts were undertaken to explain the nature and generation mechanism of the molecule, this remained elusive until 2008 when we first reported the mechanism of sfRNA generation and the important role of sfRNA in pathogenicity of WNV [18].

In this 2008 manuscript we utilized an extensive array of recombinant constructs expressing truncated lengths of the WNV genome to demonstrate that sfRNA is a product of the 3'UTR, not requiring the presence of other gRNA sequences, viral proteins or active viral replication. We identified cellular 5'–3' exoribonuclease XRN1 as the enzyme responsible for the generation of sfRNA via stalling at stem-loop (SL) structures while degrading viral gRNA. Similar to the JEV study [27], we used primer extension to map the 5'-terminus of WNV sfRNA to the beginning of SL-II structure. 

A series of deletion mutants demonstrated the importance of secondary structures within the 3'UTR in sfRNA generation with mutant viruses incapable of producing full-length sfRNA species, sfRNA1, exhibiting decreased cytopathicity in cells and attenuation of virulence in a murine model of infection.

### 1.4. Conservation of sfRNA between Different Groups within the Genus Flavivirus

**Figure 3:**
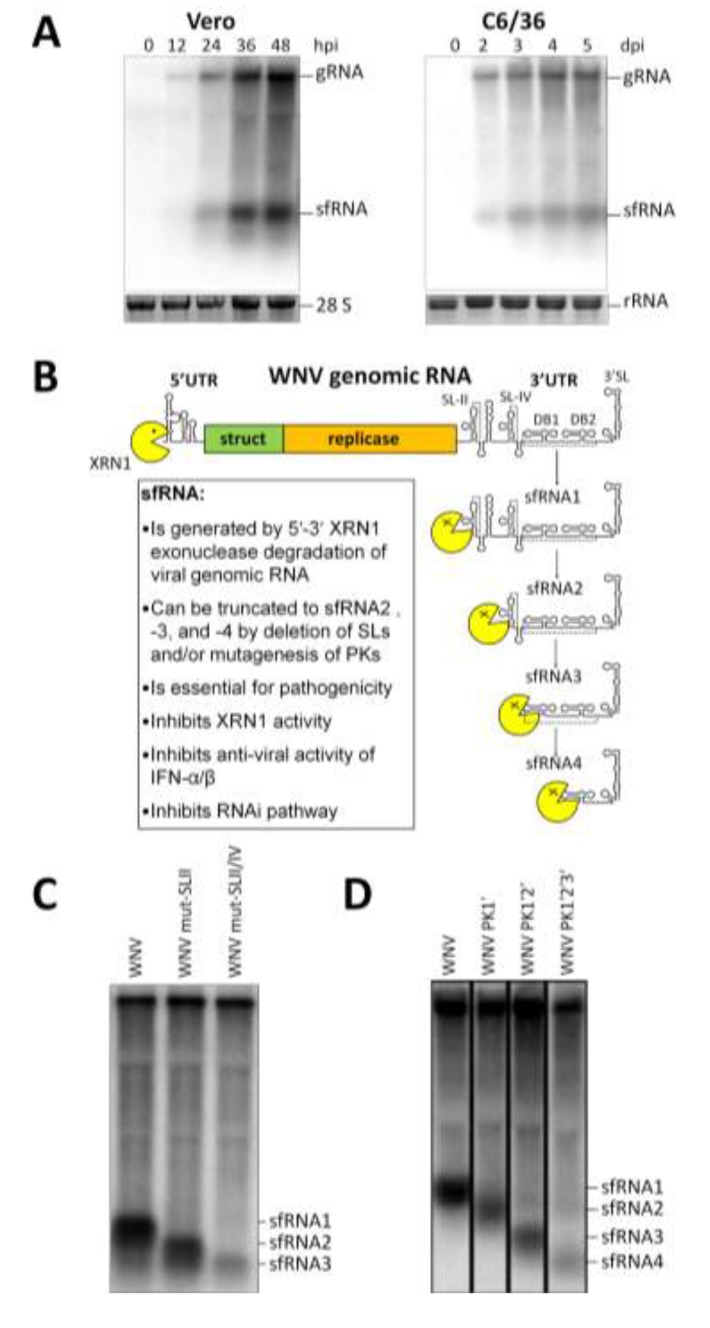
sfRNA is generated by XRN1 stalling at conserved SL and PK structures. (**A**) Northern blot detection of WNV sfRNA accumulation over the course of infection in mammalian (Vero) and mosquito (C6/36) cell lines using oligonucleotide probes complementary to the 3'SL [19]; (**B**) Schematic representation of the mechanism of sfRNA generation. Following decapping or upstream cleavage within the flavivirus gRNA, host XRN1 degrades 5'–3' stalling at the SL-II/PK1 structure and thus forming sfRNA1 and becoming inactive by association with this moiety. Mutation to delete/disrupt SL (**C**) or PK (**D**) structures leads to downstream XRN1 stalling and accumulation of successively smaller sfRNA species. Modified from Pijlman *et al.*, 2008 [18] and Funk *et al.*, 2010 [19].

The outcomes of the 2008 WNV sfRNA paper and several studies since then [18,29,30,31] were that sfRNA was demonstrated to be accumulating concurrently with genomic RNA in both vertebrate and invertebrate cells (Figure 3A) infected with members of the genus *Flavivirus* from each of the mosquito-borne (encephalitic: WNV, MVEV, Alfuy virus; non-encephalitic: Dengue virus (DENV) and Yellow fever virus (YFV)) and tick-borne (Samaurez Reef virus) groups, and absent from the genera *Hepacivirus* (HCV replicon) and *Pestivirus* (Bovine viral diarrhoea virus). Indeed, sfRNA has been demonstrated to be produced in infections with every member of the genus *Flavivirus* that has subsequently been assessed, expanding the cohort of sfRNA producers to include NKV viruses (Modoc virus (MODV), Apoi virus, Rio Bravo virus, Montana myotis leukoencephalitis virus, and Yokose virus) [32] and ISFs (cell-fusing agent virus (CFAV), Culex flavivirus (CxFV)) [33], as well as tick-borne encephalitis virus (TBEV) [34]. 

Importantly there remain at least two groups within the family Flaviviridae that have not been assessed for sfRNA production. As the most basal and distantly related member of the genus *Flavivirus* [10] it would be interesting to investigate sfRNA formation by TABV. Indeed, RNA modeling of a 241-nt sequence downstream of the predicted TABV NS5 coding sequence (partial 3'UTR; Genbank accession AF346759.1) did show the presence of a SL-II-like structure with a 4 bp-pseudoknot interaction but without downstream stemloop (Figure 2B), yet the predicted length of the resulting putative sfRNA molecule is not known until the complete 3'UTR has been sequenced. If sfRNA is experimentally demonstrated to be produced by TABV it would confirm that this moiety is characteristic of the genus, however if it is found to not be produced by this virus, this may indicate that sfRNA solely emerged as a means to facilitate infection of invertebrate hosts. This last possibility is intriguing given that sfRNA has been demonstrated to inhibit the predominant insect innate immune pathway, RNA interference (RNAi; see below) [35], and that members of the genus *Flavivirus* are the only viruses in the entire family Flaviviridae to productively infect invertebrates. The other group of viruses yet to be investigated are the members of the genus *Pegivirus* (e.g., GB virus C). Several conserved RNA secondary structures have been predicted within the 3'-terminus of pegivirus genomes, more so than that observed in hepaciviruses and pestiviruses [12]. Thus the possibility of sfRNA-like production in this genus may shed light on the evolutionary relationships of viruses in this family.

## 2. Mechanism of sfRNA Generation

### 2.1. Exoribonuclease Stalling

The unique sfRNA generation mechanism involves the efficient stalling of 5'–3' exoribonuclease XRN1 during degradation of viral genomic RNA [18,19,30,35]. XRN1 is a key enzyme in host and viral mRNA turnover [36,37] occurring in cytoplasmic processing bodies (PBs) [36] and is a classical PB marker [38]. The stalling of XRN1 in flavivirus RNA is caused by a short RNA structure of ~80 nt at the 5' end of sfRNA (called SL-II in WNV or SL-E in YFV) with high stability involving a pseudoknot (PK) base pairing interaction between the upper loop and a short sequence downstream of the SL structure (PK1, Figure 2A). Although the primary sequence is highly variable, this SL-II/PK1 structure has been predicted to be remarkably well conserved in all flavivirus strains demonstrated to produce sfRNA [17,18,31] (Figure 2B). The WNV SL-II/PK1 sequence has also been successfully used to stall XRN1-mediated degradation of herpes virus RNA [39]. The 3D structure of SL-II/PK1 remains unknown but is most likely key to fully understand its functional ability to stall and neutralize XRN1.

### 2.2. RNA Structures Required for sfRNA Production

Initial research efforts to study the 3'UTR were mostly focused on sequences at the very 3'-terminus of gRNA [13,40,41,42] while the 5'end of the 3'UTR was simply regarded as the variable domain. Yet, it is in this 5'end where one of the essential features of the 3'UTR is conserved, namely the RNA structures responsible for stalling of XRN1 [18,19,30].

The contribution of each of the SL and PK structures in facilitating XRN1 stalling has been investigated for WNV [18,19] and YFV [30]. Experiments involving disruption of these secondary and tertiary structures in the genomes of both viruses provide compelling evidence for the absolute requirement of a pseudoknot in sfRNA generation. Straightforward deletion of SL-II in WNV (FLΔSLII), mutation of the stem-loop (FL-IRA), or disruption of PK1 (FL-PK1') led to production of a smaller sfRNA species of approximately 365 nt (sfRNA2) predicted to form by XRN1 stalling at the SL-IV/PK2 (analogous in structure to the upstream SL-II/PK1) (Figure 3B–D) [18,19]. Deletion of SL-E in YFV (YFV-ΔSLE), however, led to complete abrogation of sfRNA production as determined via Northern blot using a probe complimentary to sequences in SL-B at the very centre of sfRNA [30]. An analogous phenotype was observed upon mutation of the stem-loop, or disruption of the component PK. Unlike WNV, YFV does not have a repeated SL-E structure to act as a secondary stalling point; hence the 5'–3' degradation mediated by XRN1 may no longer be prevented.

The YFV 3'UTR does contain a further two predicted PKs, forming at downstream SLs, thus these too fail to stall XRN1. This contrasts with the utility of the PK interactions in the WNV 3'UTR. Further disruption of secondary/tertiary structure in WNV via deletion of SL-II to RCS3 and CS3 (FLΔSLII-RCS3ΔCS3), disruption of SL-II and deletion of CS3 (FL-IRAΔCS3), or mutation of PK1 and PK2 (FL-PK1'2') leads to production of a smaller sfRNA species of ≈270 nt (sfRNA3) predicted to form by XRN1 stalling at DB1/PK3 (Figure 3B–D) [18,19]. An even smaller sfRNA4 produced presumably by stalling at DB2 was detected in mosquito cells infected with WNV containing combined mutations in upstream PKs 1, 2, and 3 (FL-PK1'2'3' in Figure 3B,D) [19].

In each example of deletions/mutations producing shorter sfRNA species the corresponding viruses were attenuated in regards to cytopathicity, and pathogenicity [18,19,30,43], demonstrating the importance of full-length sfRNA species, sfRNA1, to these processes. However several questions still remain to be answered in regards to the physical limitations of SL/PK interactions in XRN1 stalling. For instance, what is the minimal PK structure for stalling XRN1? What is the minimal SL-II structure? Can we learn from other viruses or different groups within the flaviviruses (e.g., ISFs)? Are these structures present in eukaryote genomes as well and, if so, do they facilitate a biological role?

### 2.3. Endogenous Production of Truncated sfRNAs

One notable aspect of sfRNA production that has received little attention is that infection with wild-type flavivirus isolates has been observed to lead to the natural production of truncated sfRNA species [19,29,30,31]. The presence of a smaller, less intense sfRNA band in a Northern blot is generally only observed occasionally for wild-type viruses and thus goes unmentioned in publications [19,29]. Such low intensity and infrequent bands likely represent occasional “slipping” of the XRN1 stalling process at SL-II. However, this occasional slipping does not appear to be the case with YFV and selected strains of DENV-2 [30,31].

Unlike the infrequent production/detection of smaller sfRNA species noted in other wild-type viruses, certain strains of DENV appear to readily produce these truncated species in infected cells [31]. This was particularly noted for the Chinese isolates of DENV-2 (strains DV2-FJ10 producing sfRNA1 and 2, and DV2-43 producing sfRNA1, 2, and 3) in infected BHK-21 cells. The three sfRNA species produced by DV2-43 were also readily observed in infected mouse brain and C6/36 mosquito cells. While the biological relevance of these truncations remains to be determined, it is important to note that DV2-43 is an isolate from an infected patient that displays no attenuated phenotypes in culture [44]. This observation contrasts with demonstrated attenuative effects of mutations in WNV leading to generation of shorter sfRNA species [18,19,43] and may therefore indicate a differential requirement for full-length sfRNA in pathogenicity/virulence of different flaviviruses.

Through the use of ^32^P-labelled oligonucleotide probes complementary to sequences at the 5'- and 3'-termini of sfRNA, a shorter YFV sfRNA2 was demonstrated to be a product of 3'-truncation in mammalian (but not insect C6/36) cell lines leading to deletion of the terminal 3'SL [30]. Notably this means of truncation appears unique to YFV as WNV does not produce this form of smaller sfRNA) [18] and the exact mechanism of this 3'–5' truncation is yet to be determined.

## 3. Cellular Localisation of sfRNA

Accurate localisation of sfRNA in infected cells has been hampered by an inability to discriminate between the 3'UTR of gRNA and processed sfRNA in infected cells. Nevertheless, the use of fluorescence in-situ hybridisation (FISH) probes to specifically detect the WNV 3'UTR (sfRNA) and NS3 gene (gRNA) has demonstrated that sfRNA forms punctuate foci distinct from the perinuclear localisation of gRNA [45]. These foci co-localise variably with XRN1 (a marker of PBs) [18] and with the stress-granule (SG) marker protein eIF3ŋ [45]. PBs are cytoplasmic aggregates that contain proteins involved in diverse posttranscriptional processes, such as mRNA degradation and RNA-mediated gene silencing [38]. The RNAi machinery is also concentrated in PBs [37]. PBs mediate the decapping and 5'–3' degradation of mRNA as part of the homeostatic turn-over of nucleic acids [36,37] and have been demonstrated to traffic some of their components to the sites of WNV replication [46]. Localisation of sfRNA to PBs is consistent with the generation of this RNA species [18], and our later studies determined that during its generation (presumably in PBs) sfRNA remains bound to and inactivates XRN1 which leads to increase in host mRNA half-lives [35] (see below). SGs accumulate during cellular response to various stress stimuli and consist of stalled translational pre-initiation complexes that act to temporarily suppress protein translation [37,47]. SGs can have diverse pro- and antiviral functions [47] and some viruses actively suppress bona fide SG assembly [48], however, the molecular interactions between viral products and SG components are only beginning to be elucidated. A biological role for sfRNA co-localisation with SGs has yet to be determined. As SGs act to sequester cellular RNA pools it is possible that sfRNA association is non-specific. It is worth noting however, that WNV has been demonstrated to actively delay the SG response by delaying early gRNA replication [49] and a functional role for sfRNA in this process cannot be discounted at this stage.

## 4. Flavivirus Replication and sfRNA

Decreased replication in mammalian and mosquito cell lines was observed for WNV and YFV mutants deficient in sfRNA [18,19,30]. A more recent study investigating the roles of sfRNA in JEV infection has revealed preliminary evidence that suggests sfRNA may play a role in viral RNA replication and/or translation [29]. Although the experimental methodology used in the JEV paper was at times highly artificial (sfRNA were transcribed *in vitro* from a T7 promoter thus are unlikely to possess an authentic 5'-monophosphate and so are unlikely to act genuinely *in vivo*), at least one key set of experiments lends credence to the authors’ hypothesis. Transfection of JEV-infected BHK-21 cells with *in vitro* transcribed antisense sfRNA at 28 h post-infection (hpi) led to a notable increase in antisense gRNA at 38 and 48 hpi compared to cells either transfected with positive-sense sfRNA or mock-transfected [29]. While the biological activity described appears genuine, it is unclear exactly how this antisense sfRNA is promoting antigenome synthesis. It may base-pair with endogenous positive-sense sfRNA and so negate a possible role in antigenome suppression. Alternatively it may displace the 5'-end of the antigenome within the replicative form to allow NS5 access and new antigenome synthesis. 

In addition, both positive-sense and antisense sfRNA were able to specifically inhibit translation of a JEV-based *Renilla* luciferase reporter minicon (*Renilla* luciferase gene incorporated in-frame after Δcapsid in a truncated JEV genome consisting of 5' and 3'UTRs, Δcapsid, Δenvelope, ΔNS1, and ΔNS5) *in vivo* in transfected BHK-21 cells. Samples exposed to control RNA derived from JEV E-NS1 sequences did not demonstrate translation inhibition. This phenotype was also observed *in vitro* during incubation with rabbit reticulocyte lysate (RRL) [29]. Unfortunately, however, the results described will need to be supported by future experiments utilizing live viral infections as these minicon investigations do not simulate the context of viral gRNA expression with absolute authenticity. Without expression of the non-structural proteins and true genomic replication, the characteristic for flavivirus infection cellular membrane environment [50,51,52] is lacking which may have profound consequences for sfRNA and translation-factor access to gRNA.

## 5. MicroRNA Production from sfRNA

The predominant innate antiviral response in insects, RNA interference (RNAi), was first identified in plants and appears to be a conserved mechanism of innate immunity and epigenetic control in eukaryote organisms [53,54]. RNAi centres on the cleavage of dsRNA into 21–30 nt long ssRNA which is then loaded into RNA-induced silencing complexes (RISC) and utilised as a homing motif for base-pairing of target sequences which are subsequently cleaved, downregulated, or upregulated. RNAi is divided into three main branches: microRNA (miRNA) which is generally involved in epigenetic control of expression, small-interfering RNA (siRNA) involved in the antiviral response, and P-element-induced wimpy testes in *Drosophila* (PIWI)-associated interfering RNA (piRNA) which is utilised primarily for the control of mobile genetic elements [53,55]. RNAi has been demonstrated to be active in the insect immune response to WNV, contributing to the evolution of viral RNA diversity [56].

Canonical biogenesis of miRNA requires a nuclear step where drosha cleaves miRNA transcript into pri-miRNA [57,58], however, mounting evidence suggests the existence of a non-canonical cytoplasmic pathway of miRNA generation [59,60]. There have been only few reports of miRNA production by cytoplasmic RNA viruses, and most of these are engineered cellular miRNA precursors inserted in the viral genome of, e.g., TBEV [59] and SINV [61].

Recently we identified the first flavivirus-derived miRNA, KUN-miR-1, in WNV-infected mosquito cells [62]. WNV sfRNA was deemed the likely source of KUN-miR-1 as expression of this RNA species from a heterologous Semliki Forest virus (SFV, an unrelated alphavirus) replicon was sufficient to lead to production of the functional KUN-miR-1 miRNA. In addition, infection with FL-IRAdCS3 mutant WNV resulted in detection of diminished amounts of KUN-miR-1, although it also coincided with diminished viral RNA replication. KUN-miR-1 was demonstrated as important for the WNV lifecycle in mosquitoes, as a specific inhibitor of this miRNA greatly reduced virus replication in mosquito cells. In addition, a host mRNA target for KUN-miR-1 in mosquito cells was determined to be zinc-finger transcription factor GATA4. WNV infection of or SFV-sfRNA replication in mosquito cells upregulated the levels of GATA4 mRNA so did the ectopic expression of pre-KUN-miR-1 alone from a plasmid DNA or from SFV replicon. Importantly, knock-down of GATA4 led to a reduction in WNV replication analogous to that observed with a KUN-miR-1 inhibitor. While the exact nature of the WNV interaction with GATA4 remains unknown, this host protein has been linked in mosquitoes to the trafficking of lipids [62]. Thus it is possible that KUN-mirR-1-induced up-regulation of GATA4 plays a pivotal role in facilitating proliferation of a lipid-rich virus-induced membrane environment known to be crucial for flavivirus RNA replication [50,51,52]. 

## 6. Host Response and sfRNA

### 6.1. Cytopathicity in Cells and Pathogenicity in Mice are Dependent on sfRNA

Early experiments to characterise the activities of sfRNA demonstrated that the cytopathic effect (CPE) generated by WNV mutants that produced less abundant, truncated sfRNAs were significantly less pronounced in Vero cells, leading to marked reduction in size or complete absence of viral plaques [18]. This loss of CPE was observed for mutants containing either deletions in SL structures [18] or disruption of PK interactions [19]. Importantly, transfection of Vero cells with a plasmid designed to produce authentic sfRNA upon XRN1-mediated degradation (pCMVβgal3') partially rescued CPE of the sfRNA1-deficient FL-IRAΔCS3 virus [18]. This phenotype can be specifically attributed to sfRNA as transfection of the control plasmid pCMVβgal did not demonstrate recovery of CPE during FL-IRAΔCS3 infection.

This reduction in CPE appears to directly correlate with attenuation of WNV virulence in a murine model of infection. Three week old mice injected intraperitoneally (i.p.) with 10,000 plaque-forming units (PFU) of both FL-IRA (sfRNA2) and FL-IRAΔCS3 (sfRNA3) failed to demonstrate symptomatic infection and remained alive by 14 days post-infection (dpi) [18]. This is in contrast to mice that received the same dose of wild-type Kunjin strain of WNV (FLSDX) which all succumbed to infection by 9 dpi. These results have been recapitulated with PK mutants: i.p. injection of three week old mice with 10,000 PFU of mutant viruses FL-PK1', FL-PK1'2', and FL-PK1'2'3' failed to induce mortality greater than 20% by 14 dpi [19] while infection of mice with wild type FLSDX virus led to complete mortality by 8 dpi in this experiment. Interestingly, all mutant viruses despite demonstrating high degree of attenuation of virulence were effective in eliciting an immune response that provided complete protection against lethal challenge with highly pathogenic New York 99 strain of WNV. Thus, mutations leading to deficiency in generation of sfRNA1 can be employed in developing effective live attenuated flavivirus vaccines. 

### 6.2. The Interferon Response and sfRNA

In order to characterise the parameters leading to the reduced pathogenicity *in vivo* observed for sfRNA-deficient WNV mutants [18,19], the potential role of the host type-I interferon (IFN-α/β) response was investigated. The IFN-α/β response pathway has been demonstrated as the most important mediator of host resistance to flavivirus infection [63,64]. Thus mutations that lead to virus attenuation are likely to affect the viral countermeasures to IFN-α/β.

Infection of wild-type (IFN-competent) mouse embryonic fibroblasts (MEFs) at a multiplicity of infection (MOI) of 1 with sfRNA-deficient FL-IRAΔCS3 led to decrease in gRNA replication and virion formation compared to FLSDX infection as measured by Northern blot and plaque assay, respectively [43]. In contrast, infection of MEFs with knock-out of the IFN regulatory factor (IRF)-3 and -7 genes (IRF-3/7^−/−^; cannot effectively produce IFN-α/β but can respond to exogenous IFN) demonstrated no appreciable difference in replication efficiency between FL-IRAΔCS3 and FLSDX viruses. 

The relationship between full-length sfRNA production and viral subversion of the host IFN-α/β response was further confirmed by complementary experiments assessing the effects of addition or neutralisation of IFN upon WNV replication. Pre-incubation of IRF-3/7^−/−^ MEFs with increasing concentrations of exogenous IFN-α followed by infection with FL-IRAΔCS3 and FLSDX viruses demonstrated a significantly higher sensitivity of the sfRNA-deficient mutant to the anti-viral activity of IFN-α. Conversely, neutralisation of the IFN-α/β receptor IFNAR1 by monoclonal antibodies during infection was able to rescue replication of FL-IRAΔCS3 mutant virus in wild-type MEFs [43].

*In vivo* experiments confirmed these results by comparing infection of wild-type C57BL/6 mice with IRF-3/7^−/−^ mice. The results demonstrated increased virulence of FL-IRAΔCS3 in knock-out mice with 80% mortality by 9 dpi with 10^3^ PFU of virus (a dose that was only able to kill 50% of wild-type mice). Additional infections of IFNAR^−/−^ mice demonstrated that FL-IRAΔCS3 was universally lethal by 8 dpi when injected with 10^3^ PFU virus (a delay of only 2–3 days compared to FLSDX). Assessment of viraemia by qRT-PCR however, demonstrated that FL-IRAΔCS3 replication, while increased in IFNAR^−/−^ mice, was still significantly reduced compared to FLSDX [43] indicating a potential partial contribution of IFN-α/β-independent host responses in controlling mutant virus replication in the mouse model of WNV infection.

Although sfRNA had convincingly been demonstrated to subvert host IFN-α/β signalling, the exact mechanism involved in this process had yet to be elucidated. IFN-α/β signalling in mammalian cells induces the nuclear translocation of phosphorylated signalling transducer and activator of transcription (STAT)-1 and -2 proteins and the upregulated expression of hundreds of antiviral IFN-stimulated genes (ISGs) [63,64]. In order to gauge the influence of sfRNA production on ISG mode of action, two well characterised ISGs known to exhibit activity during WNV replication—protein kinase R (PKR) [65,66,67,68] and RNase L [28,66,69]—were investigated for their ability to be modulated by sfRNA.

PKR has many potential roles in eukaryote cells including anti-proliferative, cell death, inflammatory, and innate immune activities. PKR recognises dsRNA of at least 30 nt in length, but optimally 70–80 nt [70]. The antiviral activity of PKR is predominantly mediated via phosphorylation of the α-subunit of eukaryote initiation factor 2 (eIF2α) at serine 51 which ultimately inhibits mRNA translation [70]. PKR^−/−^ MEFs demonstrated no observable rescue of FL-IRAΔCS3 replication compared to that observed via infection in wild-type MEFs [43], thus PKR is unlikely to be inhibited by sfRNA.

RNase L is a ssRNA-specific endonuclease activated by the binding of 2'–5'-linked oligoadenylates (2'–5'A_n_); a unique molecule produced by the 2'–5'-oligoadenylate synthetase (OAS) family of proteins upon the binding of dsRNA and stem-loops >15 nt in length [70]. RNase L is thought to exert antiviral activity via the direct degradation of gRNA as well as by cleaving host mRNAs to generate novel IFN-stimulating moieties [70]. In contrast to results obtained in PKR^−/−^ MEFs FL-IRAΔCS3 replication was partially rescued in RNase L^−/−^ MEFs, indicating a potential interaction of sfRNA with the 2'-5'-oligoadenylate synthetase (OAS)/RNase L pathway [43]. However an *in vitro* assay demonstrated that sfRNA does not associate directly with RNase L as it was unable to prevent this endonuclease from degrading WNV gRNA or other RNase L-sensitive viral RNAs. Virulence of FL-IRAΔCS3 mutant was also not rescued in RNase L^−/−^ mice further indicating that RNase L is unlikely to be a direct sfRNA target.

Preliminary evidence has also demonstrated that transfection of *in vitro* transcribed sfRNA may inhibit IRF-3 phosphorylation in JEV-infected cells [71]. The authors propose that this inhibition of IRF-3 activation may lead to a decrease in IFN-β transcription. Unfortunately, however, their experiments to assess this phenotype lacked critical controls for the efficiency of JEV infection which may itself influence IFN-β transcription and therefore further investment of research will be required to confirm and fully explore this intriguing putative role of sfRNA.

Although the possibility that sfRNA may specifically inhibit one or more proteins involved in IFN-α/β response pathway remains open, it is probably more likely that the mechanism of inhibition of anti-viral response by sfRNA is more general and is related to the ability of sfRNA to serve as a sink for cellular RNA-binding proteins that are involved in regulation of transcription and/or translation of wide range of genes involved in various cellular response pathways, including those participating in IFN-α/β response.

### 6.3. Inhibition of Host mRNA Turnover Mediated by sfRNA

The structures that lead to sfRNA generation are highly unique as they are the first RNA elements that have been shown to consistently stall XRN1 in mammalian cells. Thus the generation of sfRNA by stalling of the XRN1 enzyme is very unusual and interestingly, has an additional perhaps highly significant impact on the cell. The generation of sfRNA results in the repression of XRN1 enzymatic activity, presumably due to the slow release of the stalled enzyme from the structures at the proximal side of the flavivirus 3'UTR [35]. The repression of XRN1 by sfRNA generation occurs with both the mammalian and mosquito enzymes, thus it is likely to impact viral infection in both the host and the vector. Furthermore, sfRNA-containing substrates directly block XRN1 enzymatic activity as repression can be observed using purified recombinant enzyme and flaviviral RNA [35]. XRN1 repression, as seen by an increase in uncapped mRNAs, occurs in infections of cells with either DENV-2 or WNV. Thus flaviviruses contain a rather novel way to shut down a host cell enzyme that is likely actively trying to degrade viral transcripts during an infection.

XRN1 repression appears to have much broader impact on the cell than simply promoting the stability of flaviviral RNAs. Approximately 400 cellular mRNAs were shown to be upregulated 3X or more in a WNV infection and numerous cellular mRNAs are stabilized during flavivirus infection in an sfRNA-dependent fashion due to the apparent shut down of the entire 5'–3' RNA decay pathway [35]. The feedback of the repression of XRN1 to other factors in the 5'–3' decay pathway may be due to direct protein-protein interactions between XRN1 and decapping enzymes [72] as well as through P-bodies (which, interestingly, become disrupted in flavivirus infections [46,73]). This dramatic dysregulation of cellular gene expression at the level of RNA stability by the generation of sfRNA may significantly contribute to viral pathogenesis and immune evasion.

### 6.4. The RNAi Pathways and sfRNA

RNA viruses have small genomes carrying only a minimal set of genes required for replication but also suppression of innate immune responses of their hosts. Despite their small genome size, RNA viruses have evolved unique ways to manipulate their host cell and create a specialized intracellular micro-environment to support virus replication. For RNA viruses of insects and plants, the most potent host antiviral response they counteract is RNAi [53]. The host RNAi machinery processes the viral double-stranded RNA (dsRNA) intermediates into siRNA to subsequently target and degrade the viral RNA. It is therefore no surprise that many, if not all, insect (and plant) RNA viruses encode and produce viral suppressors of RNAi (VSR) to inhibit antiviral RNAi. While it is clear that arboviruses suffer from RNAi [74,75] and likely have strategies to dampen the detrimental effects [76], despite efforts by different research groups arboviral VSRs have not yet conclusively been identified.

Our most recent research led to the discovery of sfRNA as a suppressor of the antiviral RNAi response in different model systems [77]. The first suggestion that WNV interfered with RNAi came from experiments that showed that induced RNAi was impaired in cells harbouring actively replicating WNV replicon RNA. Next, we initiated a screen for RNAi activity of viral products (proteins, RNA) produced during WNV replication and concluded that none of the WNV nonstructural proteins could suppress RNAi, neither in mammalian cells nor in plants. However, sfRNA was the only molecule in our screen that was capable of suppressing RNAi. Subsequent experiments showed that sfRNA displayed VSR activity in both insect as well as mammalian cells and not only affected ds/siRNA-induced RNAi, but also interfered with the miRNA pathways, again both in insect as well as mammalian cells. Interference with human Dicer processing of dsRNA *in vitro* suggested that sfRNA acts as a decoy molecule upstream of the RNA-induced silencing complex (RISC). As a result from this, less (antiviral) siRNA is produced when sfRNA is present, which is in line with the observation that sfRNA enhanced the replication of a heterologous arbovirus in mosquito cells [77]. 

The observation that sfRNA is a Dicer substrate corresponds with the production of KUN-miR-1 from the 3'UTR/sfRNA that was shown to be mediated by insect Dcr-1 [62]. The relative substrate affinity of sfRNA for insect Dcr-1 in comparison to Dcr-2, which is predominantly involved in antiviral RNAi, is currently unknown, but resolving this issue could further illuminate the precise, perhaps diverse, roles of sfRNA in insects. An attractive hypothesis to be tested is whether sfRNA production is required for efficient replication of flaviviruses in the arthropod vector, but this remains to be experimentally proven. The production of sfRNA by ISFs would certainly fit in this picture. 

The exact biological activity of sfRNA as RNAi suppressor during flavivirus replication in vertebrates is still elusive, although evidence is accumulating that antiviral RNAi may exist in higher animals and humans as well [78]. In that case, sfRNA may not only feed into the endogenous miRNA pathway as we have shown [77], but could also have a profound impact on suppressing the silencing of flavivirus RNA replication in humans and other vertebrate species.

## 7. Host Binding Partners of the 3'UTR and/or sfRNA

Several candidate binding partners to the flavivirus 3'UTR have been identified via pull-down and mass-spectrometry and verified via gel shift mobility assays, immunoprecipitation, and mutational screens. Many more putative binding partners (e.g., Dicer [77]) have been inferred due to an observed functional interaction despite the lack of co-immunoprecipitation data. The binding partners variously have roles in stimulating viral gRNA replication and polyprotein translation, or function as mediators of the host anti-viral immune response. Analysis of binding partners for sfRNA specifically is distinctly lacking within the literature. Due to the conservation of sequence and structural elements between the 3'UTR and sfRNA, it is likely that many if not all of these proteins interact with this subgenomic element. Indeed due to the rapid accumulation of sfRNA it may be revealed that these proteins exert a large proportion of their functions when bound to this moiety. Table 1 summarises the identity and known functions of these 3'UTR/sfRNA binding proteins.

**Table 1:** Host binding partners of the flavivirus 3'UTR and/or sfRNA.

Protein	Origin	Function	Binds 3'UTR?	Binds sfRNA?	Method of Identification	Ref.
NS5	Virus	Polymerase 5' RNA cap	Yes, 3'SL	Likely	Infected cells Pull-down IVT ^1^ RNA	[71,79]
Capsid	Virus	Nucleocapsid	Yes	NK ^3^, Likely	Pull-down IVT RNA	[80]
NS2A	Virus	Viral RC Anti-IFN ^2^	Yes, 3'SL	NK, Likely	IVT RNA	[81]
NS3	Virus	Helicase, Protease, NTPase	Yes, 3'SL	NK, Likely	IVT RNA	[79,82]
EF1α ^4^	Host	Translation elongation factor	Yes, middle of 3'SL	NK, Likely	Infected cells, Pull-down, IVT RNA	[24,25,83]
PABP ^5^	Host	Translation initiation, SG component	Yes, A-rich regions flanking DBs	NK, Likely	IVT RNA	[23]
La autoantigen	Host	RNA chaperone, Protection from RNases	Yes, 3′SL	NK, Likely	Infected cells, Pull-down, IVT RNA	[25,84,85,86,87]
PTB ^6^	Host	RNA splicing	Yes	NK, Likely	Pull-down, IVT RNA	[25,88]
DDX6 ^7^	Host	PB component, Promote RNA degradation	Yes, DB1 and DB2	NK, Likely	Infected cells, Pull-down, IVT RNA, Quantitative mass-spec	[89]
Caprin1 ^8^	Host	Transport and translation of mRNAs, SG component	Yes, region SL-I to DB1	NK, Possibly unless binds SL-I	Pull-down, IVT RNA, Quantitative mass-spec	[89]
G3BP1/2 ^9^	Host	dsDNA or dsRNA unwinding, SG components	Yes, region SL-I to DB1	NK, Possibly unless binds SL-I	Pull-down, IVT RNA, Quantitative mass-spec	[89]
USP10 ^10^	Host	De-ubiquitination, SG component	Yes, region SL-I to DB1	NK, Possibly unless binds SL-I	Pull-down, IVT RNA, Quantitative mass-spec	[89]
FBP1 ^11^	Host	ssDNA binding protein, Influence mRNA stability	Yes	NK, Likely	Pull-down, IVT RNA	[90]
p100	Host	Transcription and RNA transport	Yes, 3'SL	NK, Likely	Pull-down, IVT RNA	[88]
IGF2BP1 ^12^	Host	Translation and mRNA stability	Yes	NK, Likely	Pull-down, IVT RNA	[88]
RBMX ^13^	Host	Pre-mRNA splicing	Yes	NK, Likely	Pull-down, IVT RNA	[88]
YB-1 ^14^	Host	Transcription regulation, Translation regulation, mRNA stability	Yes, 3'SL	NK, Likely	Infected cells, Pull-down, IVT RNA, Mass-spec	[91]
hnRNP ^15^ Q	Host	Splicing, Translation regulation, mRNA stability	Yes	NK, Likely	Pull-down, IVT RNA, Mass-spec	[91]
hnRNP A1	Host	Splicing and RNA synthesis	Yes	NK, Likely	Pull-down, IVT RNA, Mass-spec	[91]
hnRNP A2/B	Host	RNA trafficking	Yes	NK, Likely	Pull-down, IVT RNA, Mass-spec	[91]
Mov34 ^16^	Host	RNA transcription and translation, Proteasome	Yes, 3′SL	NK, Likely	IVT RNA	[92]
NF90 ^17^	Host	RNA export, RNA stabilization, Negative regulation of miRNA	Yes, 3'SL	NK, Likely	Pull-down, IVT RNA	[93]
RHA ^18^	Host	Assist NF-κB signaling, Sense dsRNA, Unwind dsRNA	Yes, 3'SL, Maybe *in vitro* only	NK, Possibly	Pull-down, IVT RNA	[93]
XRN1	Host	PB component, 5'–3' exoribonuclease	Yes	Yes	Infected cells, Pull-down	[35]

^1^ IVT = *in vitro* transcribed; ^2^ IFN = interferon signalling pathway; ^3^ NK = not known; ^4^ EF1α = elongation factor 1α; ^5^ PABP = poly(A) binding protein; ^6^ PTB = polypyrimidine tract-binding protein; ^7^ DDX6 = DExD/H-box helicase 6; ^8^ Caprin1 = cytoplasmic activation/proliferation-associated protein 1; ^9^ G3BP1/2 = GTPase-activating binding proteins 1 and 2; ^10^ USP10 = ubiquitin-specific peptidase 10; ^11^ FBP1 = far upstream element (FUSE)-binding protein 1; ^12^ IGF2BP1 = Insulin-like growth factor-II mRNA-binding protein 1; ^13^ RBMX = RNA-binding motif gene on the X chromosome; ^14^ YB-1 = Y box-binding protein 1; ^15^ hnRNP = heterologous nuclear ribonucleo-protein; ^16^ Mov34 = Moloney murine leukaemia provirus insertion-disrupted protein of 36 kDa; ^17^ NF90 = nuclear factor 90; ^18^ RHA = RNA helicase A.

## 8. Conclusions and Future Directions

**Figure 4:**
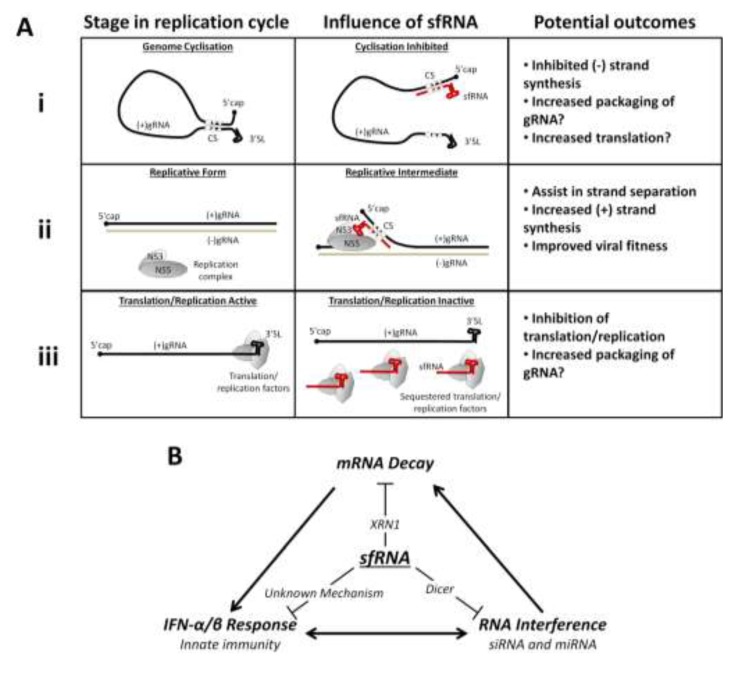
Proposed models of sfRNA interactions in viral replication and the host response. (**A**) Potential influences of sfRNA on viral gRNA replication: (**i**) sfRNA may prevent gRNA cyclisation, inhibiting (–) strand synthesis; (**ii**) sfRNA may interact via cyclisation sequences to assist in unwinding dsRNA in the replicative form at the (+) strand 5' end, this may promote increased (+) strand synthesis; (**iii**) sfRNA may competitively bind and sequester translation and/or replication factors, inhibiting translation and replication of gRNA; (**B**) Different host response pathways inhibited by sfRNA: sfRNA interaction with XRN1 disrupts host mRNA decay; sfRNA suppresses host RNAi pathways likely through interaction with Dicer; sfRNA inhibits the IFN-α/β response via an unknown mechanism. Each of these pathways cross-communicates and may influence the activities of the others.

Given their limited genome size, viruses naturally have to contain a high density of encoded information/activities. The sfRNA produced by flaviviruses certainly does not disappoint in this regard as it clearly serves multiple and highly biologically-relevant roles in virus-host interactions. The sfRNA is involved in: (i) generation of miRNA, (ii) repression of a major player in cellular RNA decay, XRN1, and (iii) binding and perhaps usurping the functions of a variety of other cellular RNA binding proteins. Collectively, these functions assist the virus in evading IFN-α/β and RNAi responses and likely contribute to the replication success as well as pathogenicity of the virus in both the arthropod vector and the vertebrate host. Based on available data, the putative models of the roles of sfRNA in virus replication (Figure 4A) and in modulating the host response (Figure 4B) can thus be proposed. Three potential mechanisms of how sfRNA may modulate different processes in the flavivirus replication (Figure 4A) include:

(1) Inhibition of antigenome synthesis: sfRNA may bind to the 5'UTR of gRNA, outcompeting intermolecular interactions with the 3'UTR and thus preventing genome cyclisation and the initiation of antigenome synthesis [20,94]. This would potentially act as a switch to increase the translation of newly synthesised positive-strand gRNA and/or packaging of gRNA into virions.

(2) Assistance in unwinding dsRNA replicative form: During the replication cycle the 5'-end of the newly completed positive gRNA must be displaced from its negative-sense partner within the double-stranded replicative form (RF). This allows the NS5 polymerase access to the 3'-end of the template antigenome [15,95]. sfRNA may assist in improving the efficiency of this event by exerting base-pairing interactions analogous to genome cyclisation to bind to the 5'UTR of positive-sense gRNA within the RF, thus displacing the completed strand and facilitating formation of the replicative intermediate [15]. As sfRNA is also likely to bind to elements of the viral replication complex (RC) (Table 1), sfRNA binding to 5' end of (+)gRNA could assist in the correct positioning of RC on the antigenome.

(3) Inhibition of translation and/or replication: sfRNA may compete with the 3'UTR of gRNA for binding to proteins of the viral RC [20,71,79,81], and/or host translation machinery [23,24,25,96]. As sfRNA accumulates over the course of an infection it may outcompete the 3'UTR and thus act as a switch late in infection that slows replication/translation to promote gRNA packaging and viral particle assembly.

The proposed model of modulation of host response by sfRNA (Figure 4B) incorporates three so far demonstrated functions of sfRNA in (i) suppression of XRN1 activity and resultant changes in stability of cellular mRNAs which leads to disruption of cellular mRNA homeostasis, (ii) subversion of IFN-α/β signalling by an unknown mechanism leading to inhibition of anti-viral response, and (iii) suppressing the utility of cellular RNAi response likely by acting as a decoy for dicer.

The crosstalk between these three cell response pathways is of particular note (Figure 4B). XRN1 inhibition may increase the half-lives of proteins that act as negative regulators of the IFN-α/β and RNAi pathways [35]. Extensive co-regulation between the IFN-α/β and RNAi pathways has also been described, with miRNAs demonstrating control over IFN-β expression [92,97] and IFN signalling in turn modulates the profiles of cellular miRNA expression [98,99]. Therefore, the incomplete rescue of sfRNA-deficient mutants in a particular knock-out cell and/or mouse line may be the result of certain redundancies and/or cross-talk between various cell response pathways affected by sfRNA. Indeed it is likely that the observed decrease in the replication of sfRNA-deficient WNV and YFV mutants in mammalian and (especially) mosquito cell lines [18,19,30] is due to an absence of the downstream effects of sfRNA antagonism of the host response (Figure 4B) leading to better cellular control of the viruses. Any intrinsic influence of sfRNA on viral replication (Figure 4A) may play a lesser role in this phenotype.

Despite rapid recent progress in identifying and characterising the mechanism of generation and functions of sfRNA, a number of interesting questions remain. The role of the sfRNA in viral interactions with the arthropod (e.g., mosquito, tick) vector needs to be investigated in more detail. Which particular nucleotide interactions in the RNA structure(s) that stalls and represses the cellular XRN1 enzyme are essential? Is this structure unique to flaviviruses or do other viruses—or even cellular RNAs—use a similar structure to stall this exonuclease to improve mRNA stability? In addition to IFN-α/β and RNAi, can sfRNA influence other RNA-related responses in the cell? Perhaps, for example, sfRNA can serve as a sink for various cellular RNA-binding proteins during infection, dysregulating a variety of pathways in which they function. The dramatic dysregulation of cellular mRNA stability due to XRN1 repression is likely to have various pathogenic consequences that need to be elucidated. From an applied perspective, sfRNA generation may represent an interesting target for the development of broad spectrum anti-flavivirus therapeutics and attenuated phenotype of sfRNA-deficient viral mutants may prove to be very useful in developing live attenuated flavivirus vaccines. Finally, with the rising appreciation of the prominence of RNA biology and post-transcriptional gene regulation in the cell, these studies also strongly suggest that the 3'UTRs of other RNA viruses be re-examined for potentially novel functions.
